# Projections of specialist physicians in Mexico: a key element in planning human resources for health

**DOI:** 10.1186/s12960-015-0061-z

**Published:** 2015-09-22

**Authors:** Gustavo Nigenda, José Alberto Muños

**Affiliations:** School of Medicine, Morelos State Autonomous University, Cuernavaca, Morelos Mexico; Independent consultant, Mexico City, Mexico

## Abstract

Projections are considered a useful tool in the planning of human resources for health. In Mexico, the supply and demand of specialist doctors are clearly disconnected, and decisions must be made to reduce labour market imbalances. Thus, it is critical to produce reliable projections to assess future interactions between supply and demand. Using a service demand approach, projections of the number of specialist physicians required by the three main public institutions were calculated using the following variables: a) recent recruitment of specialists, b) physician productivity and c) retirement rates. Two types of scenarios were produced: an inertial one with no changes made to current production levels and an alternative scenario adjusted by recommended productivity levels. Results show that institutions must address productivity as a major policy element to act upon in future contracting of specialist physicians. The projections that adjusted for productivity suggest that the hiring trends for surgeons and internists should be maintained or increased to compensate for the increase in demand for services. In contrast, due to the decline in demand for obstetric and paediatric services, the hiring of new obstetrician-gynaecologists and paediatricians should be reduced to align with future demand.

## Introduction

This article discusses the results of an exercise to project the supply of specialist physicians in Mexico. This exercise aims to propose an input for the strategic planning of the future training and hiring of specialists by the Mexican health care system. There are various models available in the literature to project the supply of health personnel, and deciding which model to use varies by each specific case. For Mexico’s particular circumstances, a key aspect is the need to produce projections for a public system that is segmented into two main sub-systems: social security and public assistance. The present exercise was designed and conducted using a “service demand” model and adjusted to the requirements of public institutions in Mexico [1]. The resulting model tried to make the best use of publicly available information from the Ministry of Health’s data system to estimate the future availability of medical specialists in the three main public health institutions (the Mexican Institute of Social Security (IMSS), the Institute for Security and Social Services for State Workers (ISSSTE) and the Ministry of Health (SSA)) which together provide services to 70–80% of the country’s population [2].

### Medical training context and links to the labour market

To adjust the supply and demand of doctors in a country is not an easy task as it requires at least two fundamental stages: a) the production of future estimations and projections and b) the discussion of these options by interested stakeholders (that is, schools, health units, hospitals and professional associations) in order to define which is the best and most feasible model taking into consideration prevailing institutional and political contexts. These exercises normally tend to reflect the preferences of interested actors who are willing to participate in planning and regulation processes [3].

In order to generate relevant policy options on how adjustments could be obtained in the future, it is important to provide background information about the current mechanisms through which physicians are trained and subsequently employed.

In Mexico, pre-graduate physicians are trained by universities who upon completion of their studies receive the degree of “generalist surgeon physician”. In recent years, there has been an increase in graduates from private schools [4]. Regulation of medical training in Mexico is weak. There are different mechanisms through which to open a school of medicine, for example, via national or state universities, state ministries of education or state ministries of health. A strict policy has existed to control the opening of new public, but not private, schools. In 2012, the National Association of Universities and Higher Education Institutions (ANUIES in Spanish) estimated there were a total of 114 medical schools in the country [5]. Currently, there is no national policy regulating the volume of new students or the quality of training. Furthermore, the volume of students trained every year is not dependent on the capacity of health institutions to employ them after graduation. Despite the existence of an accreditation process, the quality of education is not guaranteed. It is a voluntary process and only 50% of schools have been accredited, the rest being in the process of accreditation or simply not interested in acquiring accreditation status. Finally, labour market regulation is also weak. No national policy exists regarding the recruitment of health workers, salaries or incentives. Instead, each public institution defines its own policies [5].

Training programmes for specialists are always in high demand in Mexico since, upon graduation, specialists constitute an elite body of professionals who control labour market positions through their dual involvement in private and public or social security institutions. Private sector physicians may either be salaried or entrepreneurs. The majority of physicians in the latter category tends to be solo practitioners, but there is an increasing trend of working in groups or partnerships [5].

Public (Ministry of Health or SSA) and social security institutions (IMSS and ISSSTE) are the main employers of specialists. Physicians are salaried, and each institution makes its own decisions about human resources for health (HRH) policies, including the contracting of new personnel and the monitoring of performance indicators. Since it was founded in 1943, the IMSS has developed programmes to monitor productivity and improve the quality of care by their physicians [6]. In 1971, the IMSS began training family physicians to fill available slots at its new family medicine units throughout the country. Family physicians were supposed to filter the demand that was referred to specialized levels. After completing their training, graduate residents were guaranteed a position within the institution. However, 30 years later, the model was saturated and the demand for family medicine training enormously reduced. In 2012, the Interinstitutional Commission for the Training of Human Resources for Health (CIFRHS in Spanish, an intergovernmental body coordinated by the Ministry of Health) reported that out of all students who presented for the National Exam, only 11% requested Family Medicine [7]. The Ministry of Health does not hire family physicians to fill positions at the primary care level; these positions are occupied by general practitioners with no post-graduate training and senior undergraduates who by law must provide services to underserved populations over a 1-year period in order to obtain their degree [8].

Currently, the epidemiological and demographic transitions that characterize the Mexican population must be considered as health authorities attempt to respond to the dynamic health care demands of the population. The rate of population ageing has clearly increased; cardiovascular diseases, diabetes and cancers account for the highest mortality rates. One of the social security institutions, ISSSTE, serves a large segment of the elderly population. Institutions will have to make important changes to their health personnel composition, including specialist doctors, in the coming years.

### Trends in medical specialist training

The output of Mexican medical schools has fluctuated over the past 25 years. In the mid-1980s (when Mexico’s population was about 70 million), approximately 12 000 doctors graduated annually, out of a total of 60 000 enrolled medical students. This trend subsided somewhat in the 1990s due to attempts to control enrollment and increased fees at public universities. However, with the establishment of several new private schools, the number of graduates increased in the past decade: by 2005, Mexican medical schools were educating around 80 000 students and graduating 15 000 each year [5].

Specialist physicians in Mexico are predominantly trained at public sector health institutions [9]. Health institutions send the number of residency positions available for each specialty to the CIFRHS annually [9]. Having passed the National Examination for Medical Residence Applicants, administered annually by the CIFRHS, residents are assigned to available residency positions according to their exam score. For more than 30 years, all newly admitted residents have entered one of four core medical specialties: internal medicine, obstetrics and gynaecology, paediatrics and surgery. Currently, certain sub-specialties (for example, geriatrics) allow for direct residency assignment without initial training in a core specialty. In 2010, 28% of applicants (representing approximately 6000 students) were admitted to residency positions. This figure has increased in recent years due to the increased availability of residency positions [10]. Candidates who do not find a position in post-graduate training sites can search for options in the public and private sectors. The private sector has increased its demand for generalist physicians; in particular, large pharmaceutical retailers employ them to offer free consultations as a marketing strategy. However, estimations based on the National Employment Survey showed that between 2000 and 2008 around 14% of all available physicians were unemployed [5].

The projection method described above aims to adjust the demand for specialty training to existing institutional availability. Social security and Ministry of Health hospitals attract the majority of residents. However, there is no guarantee that a physician will obtain a specialist position upon finishing specialty training. On the other hand, the more specialized the physician, the greater his or her professional prestige, the higher the probability he or she can combine public and private jobs and the higher the fees for private clientele [11]. Moreover, in contrast to health systems with an integrated supply of services, in countries with fragmented or segmented health systems, patients may self-refer to private sector specialists, opting to pay out of pocket for services received. In Mexico, 50% of physicians in the specialist labour market currently practise in both public and private facilities [12].

### Planning process challenges

As outlined above, Mexico has a segmented health system, with the type of care delivery determined by whether or not a person is incorporated into the formal labour market. The Mexican government is responsible for providing medical care through its social security system comprising the IMSS, which covers those who work for private companies, and the ISSSTE, which covers workers of public sector institutions [13].

Aiming at attaining universal health coverage, the System for Social Protection in Health was created in 2003 to cover people who are self-employed or are otherwise not linked to the formal labour market. At the centralized (federal) level within the Ministry of Health, the System for Social Protection in Health finances the services delivered by state-level secretaries of health [14].

Initially, in 1983, the CIFRHS was set up to carry out the planning of human resources for the entire health system. To carry out these activities, the Commission had the support of the federal Ministry of Health, the federal Ministry of Public Education, social security institutions, representatives of universities and professional representatives, among others. Its main objective was to “identify areas of coordination between educational and health institutions for the training of human resources required by the National Health System” [15]. However, the Commission was never able to coordinate the strategic planning of HRH and major decisions about training and labour market regulations. Thirty years later, it is focused on the operation of specific tasks, most importantly the administration of the national residency exam.

The development of CIFRHS reflects to a large extent the difficulties a segmented health care system faces in estimating human resource requirements for the entire health system. Each institution hires its own personnel and defines contracting priorities according to the characteristics of the populations it serves. Recently, there has been growing interest in consolidating strategic human resource plans, but one of the main barriers to achieving strategic planning at the national level is the lack of a unit with technical capacity and enough political support to coordinate this process. These elements have shown to be major hindrances to strategic HRH planning [16]. Therefore, carrying out projections for the entire health system makes little sense; current conditions make it preferable to carry out projections by institution. Another major impediment is the lack of information and methodological tools to generate plausible scenarios that can reliably inform strategic decision-making [17].

Reliable estimates of health personnel to be hired to meet future population needs are essential to planning [18]. The ability to project specialist physicians is of great importance for a number of reasons: 1) the overall number of specialist physicians is greater than that of general and family practitioners in all three of the main public institutions, 2) specialist physicians are responsible for a large proportion of interventions at the secondary and tertiary levels of care and 3) the average number of specialist consultations per day is lower than that of general practitioners and family specialists who provide first-level care (Table 1). This issue is further explained in the “Materials and methods” section.

**Table 1:** **Office visits per doctor per day for general/family physicians and core specialty physician categories in IMSS, ISSSTE and SSA, 2000–2008**

**Year**	**IMSS**					**ISSSTE**					**SSA**				
	**General/family physicians**	**Surgeons**	**Obstetricians**	**Paediatricians**	**Internists**	**General/familyphysicians**	**Surgeons**	**Obstetricians**	**Paediatricians**	**Internists**	**General/family physicians**	**Surgeons**	**Obstetricians**	**Paediatricians**	**Internists**
2000	18.7	6.9	8.6	2.1	4.7	13.1	3.5	1.1	2.4	1.7	15.4	1.7	2.7	2.1	2.3
2001	18.6	3.2	3.1	1.2	2.9	17.0	1.3	1.2	2.7	1.7	16.0	1.7	2.8	2.2	3.4
2002	18.6	3.2	3.0	1.2	3.0	16.4	1.3	1.2	2.6	2.2	16.7	1.8	2.8	2.3	3.6
2003	17.9	3.1	2.9	1.0	2.8	18.6	1.0	1.2	2.1	1.9	17.2	1.8	2.8	2.1	3.4
2004	19.0	3.0	2.9	1.1	2.9	16.2	1.0	1.1	2.0	1.8	16.5	1.9	2.7	2.1	2.7
2005	17.9	2.3	4.0	1.0	2.1	15.3	1.4	1.1	2.2	1.5	17.4	2.1	3.0	2.4	3.4
2006	17.7	2.1	2.7	1.0	2.8	15.1	0.9	1.0	2.6	1.4	16.0	2.0	2.8	2.2	3.0
2007	18.7	2.4	2.7	1.0	2.8	15.2	1.0	1.0	2.7	1.4	14.0	1.9	2.6	2.0	2.7
2008	18.9	2.4	2.6	1.0	2.8	15.1	0.9	1.1	2.9	1.4	13.6	2.0	2.7	2.0	2.8

Note: productivity estimates include only general and specialty office visits, based on 252 workdays per year.

*Source*: Secretaría de Salud-SINAIS. Boletín de Información Estadística, 2000–2008.

*SSA* Ministry of Health, *IMSS* Mexican Institute of Social Security, *ISSSTE* Institute of Services and Social Security for State Workers.

Applying any one of the various existing health personnel projection models depends on a number of factors; perhaps most important for developing countries is the availability of information for structuring these models [3]. To that end, data limitations and convenience criteria were taken into consideration in the process of developing appropriate models to project specialist physician requirements for Mexico’s three main public health institutions.

## Materials and methods

According to McQuide and colleagues [19], there are six main approaches to HRH projections. O’Brien-Pallas et al. [20] grouped options to project the number of health personnel into three categories: 1) supply/utilization-based approaches, 2) demand-based approaches and 3) econometric approaches. Most projections are based on variations of the supply/utilization-based approach. Each approach has advantages and disadvantages, but one general observation is that there is no “right” number of personnel; at best, it is only possible to identify acceptable estimates [20]. The WHO categorizes four forecasting options: 1) the workforce-to-population ratio method, 2) the health need method, 3) the service demand method and 4) the service target method [1]. The model described below falls under the “service demand” approach. This model “draws on observed health services production rates for different population groups, applies and converts these rates to the future population profile to determine the scope and nature of expected demands for services, and converts these into required health personnel by means of established productivity standards or norms” [1].

Under the assumption that the population uses an appropriate mix of health services, our approach projects future health service needs based on the dynamics observed in the number of specialty consultations by type of specialty and institution. That being said, several recent studies and health sector evaluations carried out in Mexico have found that service utilization within health institutions is actually lower than their potential capacity to provide services [21]. It is possible to determine the number of consultations and surgical procedures a public sector doctor performs per day using the information produced by the Ministry of Health. Consultations and surgeries are considered typical outputs of a daily clinical routine, although it is clear that the latter depends on other important factors such as technology and managerial standards [22]. Based on these elements, we define productivity as the ability of an individual to generate a certain amount of expected output according to the individual’s level of training per unit of time [23]. In this particular case, it is the number of consultations and surgical procedures performed (when applicable) per day.

The Ministry of Health data presented in Table 1 show that daily office visits in public sector institutions vary according to the type of physician as well as the type of institution. General and family practitioners tend to produce an average of 17 office visits per day while specialist physicians produce only about 2.5 office visits per day – a figure which has dropped significantly in recent years. These figures could be considered low, though underreporting office visit data is a possibility because it can be difficult to measure. There is no good evidence of a tool to measure or estimate underreporting. Furthermore, considering that at the point of service delivery utilization and production are equal, we created a scenario in which production levels are adjusted to an acceptable standard. An additional benefit of this method is that it allows us to examine effective service demand, that is, population demand that translates into direct consumption of services.

For our projection estimates, we used data from 2000 to 2008 on the number of doctors hired by the country’s three main public health care delivery institutions (SSA, IMSS and ISSSTE) in each of the four core specialty areas (surgery, paediatrics, obstetrics and gynaecology and internal medicine). We only included doctors who reported working 8 h a day 5 days a week, which represents 99% of physicians in all three institutions. We linked the number and type of consultations produced by each institution during the same period, generating an indicator of the number of consultations per physician for each specialty. The number of consultations by year over the 2000–2008 period was incorporated to simulate a dynamic demand for medical consultations. We used the same process to estimate surgical procedures for the specialties of surgery and obstetrics and gynaecology.

Two projected scenarios were subsequently generated from this 9-year series on the number of specialists and specialty consultations: 1) a scenario-denominated “inertial” which projects the growth trend of each type of physician specialty area and 2) a scenario considering the current productivity outputs of consultations and surgical procedures per doctor per day, which adjusts for productivity according to expert opinion findings (explained below) for each specialty. Ninety-five per cent confidence intervals for adjusted projections are shown in Table 2. Furthermore, we decided to incorporate the number of speciality consultations as a means to analyse the differential behaviour of the forecast series, given the potential modification of the control parameters and as a way of carrying out a sensitivity analysis.

**Table 2:** **Projected number of specialists required by IMSS, ISSSTE and SSA, 2030 (adjusted scenarios)**

**Year**	**Obstetrician-gynaecologist**			**Paediatrician**			**Internal medicine specialist**			**Surgeon**		
	**Scenario**	**CI low**	**CI high**	**Scenario**	**CI low**	**CI high**	**Scenario**	**CI low**	**CI high**	**Scenario**	**CI low**	**CI high**
	IMSS											
2010	2213	2172	2231	588	532	642	987	971	1004	4000	3526	4474
2015	2256	2194	2296	502	444	557	1117	1097	1138	4850	4363	5337
2020	2299	2216	2361	415	356	472	1247	1223	1273	5701	5198	6203
2025	2342	2237	2426	328	267	387	1376	1349	1407	6552	6030	7072
2030	2386	2258	2491	242	179	303	1506	1475	1542	7402	6861	7942
	ISSSTE											
2010	611	537	688	263	243	284	407	372	431	594	426	845
2015	739	501	991	247	224	271	458	399	505	595	424	844
2020	868	458	1302	230	205	258	509	424	583	590	422	843
2025	996	413	1614	214	186	245	559	447	661	554	420	842
2030	1125	369	1927	197	166	233	610	470	740	553	418	841
	SSA											
2010	2934	2709	3150	1574	1512	1639	1126	879	1372	2858	2611	3105
2015	3539	3308	3761	1903	1838	1969	1526	1272	1779	3455	3201	3708
2020	4144	3906	4374	2231	2164	2299	1927	1664	2187	4051	3788	4312
2025	4750	4503	4987	2559	2490	2630	2327	2055	2596	4648	4375	4918
2030	5355	5099	5602	2887	2816	2960	2727	2444	3007	5245	4961	5524

Note: Productivity estimates include only general and specialty office visits, based on 252 workdays per year. Confidence interval set at 95%.

*Source*: Secretaría de Salud-SINAIS. Boletín de Información Estadística, 2000–2008.

*SSA* Ministry of Health, *IMSS* Mexican Institute of Social Security, *ISSSTE* Institute of Services and Social Security for State Workers.

Suggested average values were obtained from broad consultation with clinicians and health unit managers. A group of 15 participants was asked to consider the suggested productivity by specialist category, taking into account institutional environment (for example, lack of appropriate resources, time dedicated by doctors to non-clinical procedures, vacations and other non-labour days) as well as the time elapsed between first and subsequent consultations (http://salud.edomexico.gob.mx/html/transparencia/informacion/manualprocedimientos/mprocedimientos/MP_CONSULTA_EXTERNA.pdf; (última consulta: 26/05/2014)). The group was given two opportunities to provide average estimates in order to arrive at a consensus. Final productivity figures do not intend to reflect the ideal number of office visits and surgical procedures but instead those that could be performed according to existing resource restrictions.

Therefore, daily productivity adjustments were incorporated using the following values: a total of eight office visits for paediatricians and internists, a total of two surgical procedures and two office visits for surgeons and a total of one procedure (either a surgical procedure or a delivery) and four office visits for obstetrician-gynaecologists (ob-gyns). Depending on the institution, for paediatricians and internists, the adjusted figures represent between 2.7 and 8 times the current number of consultations. For surgeons, the figures remained roughly the same, and for ob-gyns, they were two to four times the current number.

The projections were made using Zaitun Time Series software (http://www.zaitunsoftware.com/), which was designed for statistical analysis of time series data. Rising trends were observed upon initial exploration of the 2000–2008 time series. Therefore, to allow for robust forecasting and to enable modelling of the time series based on the ncreasing trend, the following exponential smoothing techniques were incorporated into the analysis.

To build the adjusted forecasting scenarios, the smoothing technique was based on the time series trends of both the four categories of specialist doctors (surgeons, internists, ob-gyns and paediatricians) as well as the number of consultations and surgical procedures. When the series showed a linear trend – where the level and the growth rate could be changing without a seasonal pattern – the exponential smoothing estimate used the following equations:

1 $$ {S}_n=\alpha {Y}_n+\left(1-\alpha \right)\left({S}_{n-1}+{T}_{n-1}\right) $$

2 $$ {T}_n=\gamma \left({S}_n-{S}_{n-1}\right)+\left(1-\gamma \right){T}_{n-1} $$

3 $$ {\widehat{Y}}_{n+m}={S}_n+{T}_nm $$

Equation (1) estimates the smoothing value *S*_*n*_ of the trend in the previous period *T*_*n*__ − 1_ added to the last previous value *S*_*n* − 1_, where *α* is a smoothing coefficient between 0 and 1 and *Y*_*n*_, the real value for the series over the *n* period. Equation (2) estimates the value of the *Tn* trend of *S*_*n*_, *S*_*n*__ − 1_ and *T*_*n*__ − 1_, where *γ* is the trend’s smoothing coefficient between 0 and 1. Finally, equation (3) (backwards prediction) is obtained from the *Tn* trend, multiplied by the increase in the following forecasting period, *m*, and adding base value *Sn*. For initial values *S*_0_ and *T*_0_, the square minimums method was used. The estimation of the *S*_0_ value is the value of the linear estimate constant, while *T*_0_ is the slope value.

If the time series of specialists or consultations/surgeries also contained a trend, then Holt’s double exponential smoothing method was used. However, if the time series presented a seasonal component then, the Holt-Winters triple exponential smoothing method was used. This method is based on three smoothing equations: stationary component, trend and seasonal.

Smoothing equation:

$$ {\mu}_n=\alpha \frac{y_n}{S_{n-1}}+\left(1-\alpha \right)\left({\mu}_{n-1}+{T}_n\right) $$

Trend smoothing:

$$ {T}_n=\gamma \left({\mu}_n-{\mu}_{n-1}\right)+\left(1-\gamma \right){T}_{n-1} $$

Seasonal component:

$$ {S}_n=\beta \frac{Y_n}{\mu_n}+\left(1-\beta \right){S}_{n-1} $$

Predictive value:

$$ {\widehat{Y}}_{n=m}=\left({\mu}_n+{T}_nm\right){S}_{n-l+m} $$

Where *μ*_*n*_ is the new smoothened attenuated value, *α* is a smoothing coefficient between 0 and 1 and *Y*_*n*_ is the new observation or real value of the series at moment *n. γ* is the attenuation constant of the trend estimation and takes values in the 0 to 1 interval; in other words, the estimation of the trend. *β* is the attenuation constant of the stationary estimation and takes values in the interval (0, 1), *m* is the number of periods to be forecasted in the future, *l* is the seasonal length (by month or year quartile), *T* is the trending component, *S* the seasonal adjusting factor and *Ŷ*_*n*__+__*m*_ is the forecasting value for the following *m* period.

The results were verified using different model parameters by means of an algorithm incorporated in the programming software Grid Search, which was useful in observing the forecasts’ mean square errors. For the election of initial parameters, a grid search of 100 results was carried out. As a result, less weight is given to the most remote observations and greater weight to more recent observations. For each case, we chose to use the model with the smallest mean absolute error (MEAN).

## Results

Projection results are presented according to four graphs. With the exception of surgeons required by the IMSS, we observed productivity-adjusted projections that are well below the inertial growth trend projections for all categories of specialist physicians.

**Figure 1:**
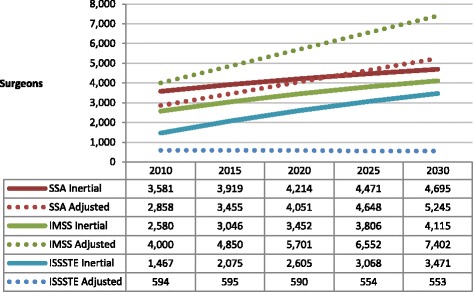
**Projected number of surgeons required by IMSS, ISSSTE and SSA.** (Inertial and productivity adjusted scenarios). Source: Secretaría de Salud-SINAIS. Boletín de Información Estadística, 2000-2008. Annotations: SSA = Ministry of Health; IMSS = Mexican Institute of Social Security; ISSSTE = Institute of Services and Social Security for State Workers.

Figure 1 shows the required number of surgeons projected for 2030 in each of the three institutions. Due to the projected increase in demand for surgical procedures and post-operative visits, 2030 projections for both IMSS and SSA show an increased number of required surgeons. However, productivity adjustments suggest that ISSSTE should hire fewer surgeons than the estimate based on what inertial hiring tendencies suggest (should these specialists succeed in adhering to an average of two surgeries and two office visits per day). In fact, according to the adjusted scenario for 2030, ISSSTE will not require any additional surgeons. Both SSA and IMSS should hire more surgeons than the number projected by the inertial hiring tendency, but IMSS requirements are far higher due to the imminent retirement of surgeons in the coming years.

**Figure 2:**
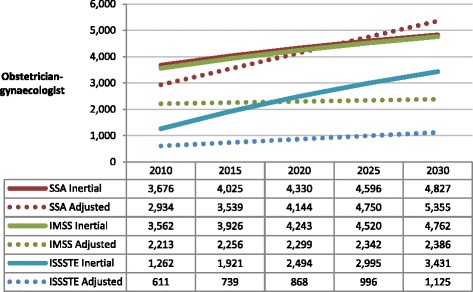
**Projected number of obstetrician-gynaecologists required by IMSS, ISSSTE and SSA.** (Inertial and productivity adjusted scenarios). Source: Secretaría de Salud-SINAIS. Boletín de Información Estadística, 2000-2008. Annotations: SSA = Ministry of Health; IMSS = Mexican Institute of Social Security; ISSSTE = Institute of Services and Social Security for State Workers.

In the case of ob-gyns, as shown in Figure 2, productivity values were adjusted to one procedure and four office visits daily. To avoid simulating excess caesarean section rates in all institutions (particularly at ISSSTE) [22], the number of daily procedures including both non-surgical procedures including vaginal deliveries and surgical procedures including caesarean sections was left at one per doctor per day.

The behaviour of the projections is similar in the social security institutions IMSS and ISSSTE but different at SSA. Only in the latter institution does the adjusted projection surpass the inertial projection, meaning that SSA should increase the hiring of these specialists in the future. However, differences between both projections are minimal. At IMSS and ISSSTE, inertial trends will lead to a potentially unnecessary increase of ob-gyns by the year 2030. At that point, the difference between the inertial and the adjusted number of specialists required would be 1.9 times higher for IMSS and 3 times higher for ISSSTE. By 2030, the number of ob-gyns at ISSSTE would be 28% less than IMSS and SSA, but ISSSTE would only be providing services to less than 10% of the Mexican population. According to the adjusted projection, the number of ob-gyns required for 2030 at IMSS and ISSSTE would be similar to the existing number for 2010. Furthermore, the volume of all gynaecological and obstetrical interventions (consultations, deliveries and surgeries) has fallen in recent years (except for SSA), alongside an increase in the number of ob-gyns entering the field. These factors drive the differences between inertial and productivity-adjusted projections.

**Figure 3:**
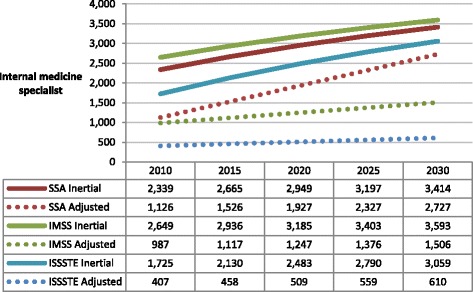
**Projected number of internal medicine specialists required by IMSS, ISSSTE and SSA.** (Inertial and productivity adjusted scenarios). Source: Secretaría de Salud-SINAIS. Boletín de Información Estadística, 2000-2008. Annotations: SSA = Ministry of Health; IMSS = Mexican Institute of Social Security; ISSSTE = Institute of Services and Social Security for State Workers.

Figure 3 presents projections of internal medicine specialists required by the three institutions. Population demand for internal medicine has increased over time, and this momentum will probably continue into the future since the population is expected to present more cases of diabetes, hypertension and associated chronic conditions. Trends in internal medicine specialist projections are similar across all three institutions: in all cases, there is a substantial gap between the inertial scenario and the productivity-adjusted scenario (with an ideal productivity of eight office visits per day), with the maximum difference observed in the year 2030 for ISSSTE.

For IMSS, the relative difference observed between the inertial scenario and the productivity-adjusted scenario is reduced from 2.6 to 2.3 times between 2010 and 2030. This implies that, during the next 20 years, the growth of demand for internal medicine consultations will increase slightly faster than the institutional availability of internists. The gap between internal medicine specialists required by the ISSSTE is ever widening over the forecast period: the inertial projection is 4.2 times greater than the productivity-adjusted projection in 2010 and will impressively increase to 5 times greater in 2030. On the other hand, the relative gap between the two projection estimates in favour of the inertial scenario is slightly higher by the end of period for the SSA, from 0.1 times more internists in 2010 to 0.2 in 2030. For ISSSTE in the adjusted scenario, the amount of internists hired today will remain roughly the same in order to produce the required consultations by 2030, and IMSS will require moderate growth. Unlike the social security institutions, the number of internists will have to increase more than 100% to cover the demand for consultations at SSA.

**Figure 4:**
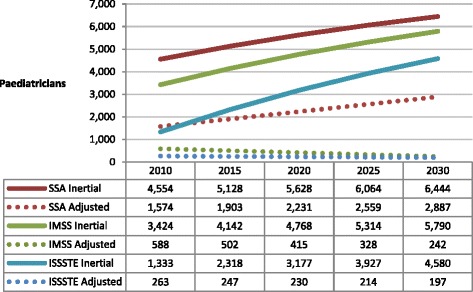
**Projected number of paediatricians required by IMSS, ISSSTE and SSA.** (Inertial and productivity adjusted scenarios). Source: Secretaría de Salud-SINAIS. Boletín de Información Estadística, 2000-2008. Annotations: SSA = Ministry of Health; IMSS = Mexican Institute of Social Security; ISSSTE = Institute of Services and Social Security for State Workers.

Finally, as with demand for gynaecological and obstetric care, demand for paediatric care has been declining in recent years due to reduced fertility rates in the Mexican population. Paediatrician projections are displayed in Figure 4.

In inertial scenarios, we observe an increase in the hiring of more paediatricians for all three institutions. However, the reduced tendency in demand results in diminishing productivity-adjusted estimates for the number of paediatricians needed in the future for both IMSS and ISSSTE. In the SSA, this particular trend is not observed; in fact, the productivity-adjusted growth projection is quite similar to the inertial trend. In the IMSS, the fall in demand is so dramatic that the adjusted projection for 2030 is 23.2 times lower than the inertial projection. For ISSSTE, the difference between inertial and productivity-adjusted scenarios in the projected number of paediatricians required in 2030 is also 23 times higher.

## Discussion

The HRH literature points out that when it comes to projections, there is no perfect model appropriate to all situations. For many countries, the major limiting factor is the unavailability of information that can serve as reliable projection input. Hence, different approaches have been developed for different countries. For example, Kurowski et al. [24] developed a model to forecast human resources in Tanzania based on three elements: services quantity, tasks and productivity, which considers the possibility of projecting human resources to expand the coverage of Millennium Development Goals interventions. In Ethiopia, a different approach was taken to carry out an overall health system forecasting of human resources. It took into consideration health service location, staffing levels, population growth and economic growth [23]. Despite their different methodological approaches, both experiences show that countries had to make enormous efforts to increase the amount of human resources to match requirements.

In Mexico, the availability of information on the health system has improved in recent years to the level that it is feasible to carry out different types of projection exercises. A pioneering exercise done by Myers in 1970 used data on physicians in relation to population, which were the only type of data available at that time [25].

Given the information we had available, we elected to make projections of specialist physicians for public sector health care institutions in Mexico under a “service demand approach” [19,20], with productivity adjustments applied to generate desired scenarios stemming from increased service production capacity in the workforce. There are several limitations to our approach that must be considered when interpreting the results. First was the need for data to incorporate multiple variables that could be coherently matched. We attempted to reduce this limitation by using the data made available by the Ministry of Health. A second limitation is that the method used in some cases produces 95% confidence intervals close to the precise estimates stemming from the initial adjustment of data. Therefore, estimates should be interpreted with caution, and as any other projection exercise, estimations should be revised at any time, if new data is available. A third limitation is the assumption that the population actually needs currently offered services and is correctly using the health care options they have at hand, which contrasts with the low production of services identified by the study. A fourth specific limitation is that our models focused only on those physicians who were already hired by the institutions and did not consider the supply of new specialists.

In our model, a key variable was production of services by physicians, but defining “products” was not a straightforward decision. “Products” were associated with the type of specialty practised by the physician. The production capacity of individuals in health institutions is, of course, mediated by different kinds of factors [26]. Different types of associated factors depend on the type of product to be generated. For example, general or family practitioners are less dependent on technology and infrastructure in a health facility than are surgeons. Among all the specialists included in this study, it follows that internal medicine specialists and paediatricians are conceivably less dependent on available hospital technology than surgeons and ob-gyns.

The production of services is also an expression of the population demand that health care institutions are capable of meeting. Although other projection models use estimated demand generated directly from the population [20], for our study, it was not possible to obtain information at that level; as such, our model was based on the demand met by the health-care-providing institutions included in the exercise. This could be considered another limitation of this approach as it does not count unmet demand, and therefore, projections entail a degree of inefficiency in the institutional response.

Policy-makers should be aware of these limitations if using the results provided by our models for planning purposes. Projection models represent tools that can offer a perspective of future trends according to a broad variety of parameters. These parameters can change over time, and models should be re-adjusted to provide the most accurate picture according to new parameters. In our case, beyond the trends described, it is clear that policy-makers should consider productivity of physicians as one parameter to adjust in order to increase efficiency and have a better sense of the future need for specialists.

Mexican policy-makers should be aware that the majority of the adjusted scenarios generally follows a different trend compared to inertial scenarios. This means that the inertial scenario would eventually lead to serious distortions in the recruitment and productivity of new specialists and that this would require, sooner rather than later, strong policy decisions. In those cases where inertial and adjusted trends are similar, no great changes are required, but the process should be monitored.

One of the benefits of this approach is that it makes productivity rates and their implications apparent: low productivity points to the existence of inefficiencies in the use of available human resources, while extremely high productivity suggests the need to step up the number of available human resources via increased hiring. Projections were also adjusted by the rate of retirement. This factor has recently been highlighted by political actors as a major justification to increase the number of specialists doctors trained and hired by institutions. Retirement rates reduce the projected availability of doctors, but this effect is offset by the number of new specialists trained every year. In other words, adjusting for retirement rates would allow for more precise projections. In fact, as retirement rates have been growing in recent years, future projections should consider updated retirement rates.

Projection results clearly show that the Mexican health system has a major problem with specialist productivity. Productivity rates are decreasing, meaning that the overall demand for specialist consultations and surgeries could be dealt with by a fraction of the specialists that are currently working in public institutions. As in many other countries, specialist doctors in Mexico are highly capable of influencing technical, clinical and managerial processes [27]. It is also common for this influence to yield group, but not necessarily institutional, benefits. It is likely that behind the low observed rates of specialist physician productivity is the ability of doctors to exert some degree of control over the definition and application of managerial guidelines in health units [28]. Human resource productivity is a key factor health sector planners should be concerned about [29]. Productivity of Mexican doctors is low by international standards according to a recent report by the Organisation for Economic Co-operation and Development on the amount of consultations per doctor per day across member countries. Mexico shows the lowest average of all member countries with 2.9 consultations in 2010. In the same year, Chile achieved 3.3 consultations per day and Turkey 7.3. As further reference, in the same year, Korea reached the highest number of consultations with 12.9 [30].

In practically all cases, the productivity adjustment leads to a projection of physician requirements well below the inertial, non-adjusted projections. In this regard, tendencies in population demand dynamics in recent years cause two trends to stand out in the projections. Firstly, due to a decrease in the fertility rate – which fell from 4.8 live children per woman in 1980 to 2.4 in 2000 – demand for gynaecological and obstetric, as well as paediatric care, clearly begins to decline. At the same time, demand for internal medicine and surgical procedures is increasing. In particular, demand for hospital care for diabetes and hypertension, which fall under the internal medicine specialty area, will likely experience further growth in coming years [31].

Institutions must respond quickly regarding the hiring of health personnel based on these trends. However, it is entirely possible that current and future hiring decisions do not consider the importance of these trends [32,33]. The data show that between 2000 and 2005, the IMSS hired a substantial number of surgeons, ob-gyns and paediatricians to contend with future demand. Despite the smoothing applied to inertial projections, they exceed the adjusted projections of the number of those specialists required in all cases, with the exception of surgeons. This means that the IMSS does not consider the role of current productivity in its hiring decisions, despite the data compiled and published by the Ministry of Health which led to our findings. Similarly, the SSA shows significant growth patterns in the hiring of ob-gyns and paediatricians, but not internal medicine specialists nor surgeons. ISSSTE showed the lowest productivity rates of all three institutions, particularly in the most recent years. ISSSTE is a state institution dominated by political interests, especially when it comes to the very influential role of the union. Specifically, the union has resisted the introduction of new managerial models that promote the application of efficiency standards.

Other elements probably exert an influence on hiring decisions as well. The inertia typical of specialist physician training in Mexico puts great pressure on institutions to hire newly trained specialists upon completion of training, since the institutions themselves are the ones who train them. Institutions invest significant resources in this training, and electing not to retain newly trained specialists seems like a waste of resources or an investment on which other institutions (whether public or private) can capitalize at very low cost.

The results of this study have important implications in the context of the integration of the Mexican health system [34]. Health personnel and particularly specialist physician planning must be carried out in relation to the goals the system seeks to achieve as a whole, not simply according to the target each institution sets for covering its respective population. Tightening up productivity is important for all institutions and also contributes to the goal of improving the entire system’s level of efficiency. Instead of specialist physicians participating in a private market characterized by open supply and demand – in which the user invariably ends up paying out of pocket – Mexican institutions should create mechanisms under which users are financed by an insurer, like in Great Britain, thereby giving physicians an incentive to care for patients as part of an institutional consultation and not as part of his or her private practice [35].

Another important aspect of a systemic perspective is that Mexican health institutions have been expanding investment in promotion and prevention activities to respond to the increasing demand of chronic disease. Ultimately, this investment will pave the road to strengthen the shift towards a primary health care model where family physicians play a more relevant role. Mexican institutions today hire more specialists than family physicians, and this balance needs to shift in the coming years.

As explained in the opening sections, production and recruitment of doctors are barely regulated. Although educational and health institutions as well as professional boards have been working towards strengthening the regulatory framework, the road has been rocky [5]. Clearer regulations are needed to open new medical schools – and only when properly justified – to improve the quality of education and establish a number and mix of newly trained specialists that is compatible with health institution requirements. Health institutions urgently need to regulate productivity and improve quality of care and the balance between the number of specialists and family physicians.

The results presented here call for the use of this type of exercise in strategic planning processes that involve estimating the response to population needs by institutions which – while they may continue to be structurally separate – are capable of producing a coordinated supply of health personnel while actively engaging providers through incentives that promote service production in line with institutional goals.

## Conclusions

Specialist doctors in Mexico currently represent more than 50% of doctors hired in public institutions. This abundant presence is a reflection of a health care model dominated by hospital care services and the lack of human resources for health planning. Projections comprise a key tool to understand future consequences of training patterns and the capacity of institutions to hire and get the most out of their labour capacity. Projections, however, should always be designed to generate information within a specific country’s context and a certain minimum availability of data. In the Mexican case, we found that by incorporating productivity and retirement rates it was possible to correct trends that otherwise would be unrealistic. In particular, increasing productivity is a key policy to implement since, unlike other factors, it is under institutional control. It is clear that health institutions need to undertake planning based on evidence to reduce the incorporation of paediatricians and gynaecologists and increase that of internists and surgeons. Even more important is to redefine the role of hospitals and specialists within the context of an integrated primary health care strategy that would allow the system to successfully tackle the consequences of the epidemiological transition [36].
